# The Book World of Medicine and Science

**Published:** 1895-11-09

**Authors:** 


### The Book World of Medicine and Science.

The Theory and Practice of Counter-Irritation. By H. Cameron Gillies, M.D. (London: Macmillan and Co. 1895. 236 pages. Price 6s. net.)

It is a good thing now and again—not perhaps every day, but as an occasional exercise—to take up some one topic connected with one's professional work, to look at it from every point of view, to put the different questions which arise thereon into literary form, to discuss with good humour the *pros* and *cons.*, to find out what old authors have said about it and compare their sayings with the pronouncements of the newer schools, and finally to make up one's mind whether in it there is aught that may be good and practical. All this has been done for the subject of counter-irritation by Dr. Gillies, who, in the book before us, has taken up what some might call the comparatively small subject of counter-irritation, and shown us how interesting it is possible to make even blisters and mustard poultices by aid of a readable literary style and a scientific spirit of inquiry.

The book is divided into two portions—first, the historical and critical; second, the practical. Of these two the first is in many ways the best. Destructive criticism is often pleasant reading, whereas the practical teaching of a method is apt to become somewhat dull, and, moreover, in turn gives openings for objections, and in this the book in question is by no means different from many others. Nevertheless, there is much which is interesting in this practical portion.

The essential action of a counter-irritant is stated to be to quicken the circulation of the part to which it is applied, and towards the point of application. It is on this action that almost all beneficial results from this mode of action follow. In this way it is pointed out that in the aged repair may be so slow or inefficient as to need the aid of stimulation even in vascular parts; and that injuries to parts not well supplied with blood, even in the young and vigorous, will repair and heal very much quicker with the use of a stimulant irritation.

This view of the *modus operandi* of counter-irritation receives its most forcible illustration from its efficacy in the relief of pain. Pains which are the result of gross destructive injury of parts or of progressive disease or violence are not relievable by counter-irritation; but neuralgias, so called, in which no morbid condition can be discovered beyond what the persistent neuralgia itself induces, and idiopathic inflammatory states, generally call for counter-irritation. These two classes of disease comprehend nearly all forms of pain to which this treatment is applicable, and it is suggested that in all these painful states there is defective local nutrition, and that counter-irritation has its good effect in the simplest and most direct manner, by bringing more blood to the part, and so improving nutrition and raising the vitality.

Special emphasis is laid upon the fact that, whenever counter-irritation is used for the purpose of accelerating local nutrition, this local benefit is at the expense of the rest of the body, and that it is, therefore, necessary in such cases to sustain the general nutrition of the patient to the utmost. "Pain is the prayer of a part for food." The local increase of the blood supply by means of counter-irritation must then always be accompanied by an improvement of the general nutrition if real good is to be done.

One of the most interesting illustrations of the utility of this method of treatment in the management of idiopathic inflammatory states is derived from the treatment of acute rheumatism by the application of blisters, as recommended by Dr. Herbert Davies, a method which at the present time is far too much overlooked, probably in consequence of the comparative ease with which pain and discomfort are removed by drugs of more recent introduction. A final chapter gives a very practical summary of the various means used for the production of counter-irritation, giving useful suggestions as to the choice of one or other in the various cases met with in daily practice. Altogether, the book is a curious mixture of pure theory and of the teachings of practical experience, and is certainly well worth reading by all who take a wide and philosophical view of the problems presented to them in their daily work.

A Baneful Popular Delusion on the Subject of Motherhood. By William Woods Smyth. (London: Simpkin, Marshall, Hamilton, Kent, and Co. 1895. Price 6d.)

The writer of this pamphlet has arrived at a conclusion which must be very satisfactory to all mothers, but not by any means so pleasing to their maiden sisters. He declaims with great energy against the popular notion that for a woman to become a mother is to entail upon her a very great and grave personal loss. Such an idea is, he says, entirely untrue, the very opposite holding good, for "the mother, through living union with her child, is made to partake again of that stream of immortality of which Professor Weissman discourses, with the effect of having restored to her stock of life more than she gave." How this happens we are told with the greatest frankness, for we are told that "in the development of the babe quantities of germinal matter, becoming unlocked in the course of that development, enter the circulation of the mother and more than provide for all the outlay she may be called upon to make on behalf of her offspring." How the poor birds and fishes which are not able to re-absorb this vital energy during fœtal growth manage to keep up their power we are not told, but we are assured that, while many mothers are stronger in health and constitution than their maiden sisters, such results of maternity would be even more general were it not for the universally accepted, but false, suggestion that to become a mother a woman must also become an abridged and wasted specimen of humanity, the truth being that "we do not lose but gain by accepting the natural commission which God has given to our race." All this is very satisfactory, and must be especially so to the tired, neglected, and worn-out specimens of the genus wife, who, after bearing a dozen children, spend their after years in being their drudge, for they indeed must sadly want the additional life energy which our author fancies is given to every mother.

